# Increased rate of injuries to the anterior cruciate ligament in amateur soccer players after the COVID-19 pandemic lockdown

**DOI:** 10.1007/s00402-024-05531-y

**Published:** 2024-09-14

**Authors:** Clemens Memmel, Werner Krutsch, Johannes Weber, Lorenz Huber, Maximilian Kerschbaum, Markus Rupp, Volker Alt, Dominik Szymski

**Affiliations:** 1 Department of Trauma Surgery, University Medical Center Regensburg, Franz-Josef-Strauss Allee 11, 93053 Regensburg, Germany; 2 FIFA Medical Center of Excellence, University Medical Center Regensburg, Regensburg, Germany; 3 Department of Pediatric Surgery and Orthopedics, Hospital St. Hedwig, Barmherzige Brueder Regensburg, KUNO Pediatric University Medical Center, Regensburg, Germany; 4 SportDocs Franken, Nuremberg, Germany

**Keywords:** Corona virus, Sports injury, Sports medicine, ACL, Knee injury, Team sports, Lockdown, Pandemic

## Abstract

The COVID-19 pandemic led to the discontinuation of soccer activities at all levels of play. Injuries to the anterior cruciate ligament (ACL) are still one of the most severe injuries in soccer. The aim of this investigation was to analyze the changes in the incidence and mechanism of ACL injuries in professional and amateur soccer before and after the lockdown period. Injury data were prospectively collected using the database ‘ACL registry in German soccer’. Between 2019 and 2021, 10 matches in professional, semi-professional, and amateur leagues before and after the lockdown were analyzed for the incidence and mechanism of ACL injuries. Data were collected using standardized and recently published study methods. Mean incidence of the 84 ACL injuries recorded during the study period was 0.083 injuries per 1000 h of exposure before and 0.079 injuries per 1000 h of exposure after the lockdown period (*p* = 0.699). In amateur soccer, the incidence of ACL injuries increased significantly from 0.058 injuries per 1000 h of exposure before to 0.128 injuries per 1000 h of exposure after the lockdown period (*p* = 0.026), in contrast to professional (*p* = 0.436) and semi-professional (*p* = 0.802) soccer. The predominant mechanisms of ACL injuries were non-contact injuries (pre-COVID: 59.1%; post-COVID: 57.7%) and indirect contact injuries (pre-COVID: 31.8%; post-COVID: 30.8%). The absence from training and match practice in German soccer during the COVID-19 lockdown led to a significantly increased ACL injury rate in amateur leagues, while in professional and semi-professional soccer no differences were reported. The mechanism of ACL injuries did not change after the lockdown period.

*Level of evidence*: II.

**Supplementary Information:**

The online version contains supplementary material available at 10.1007/s00402-024-05531-y.

## Introduction

**Fig. 1:**
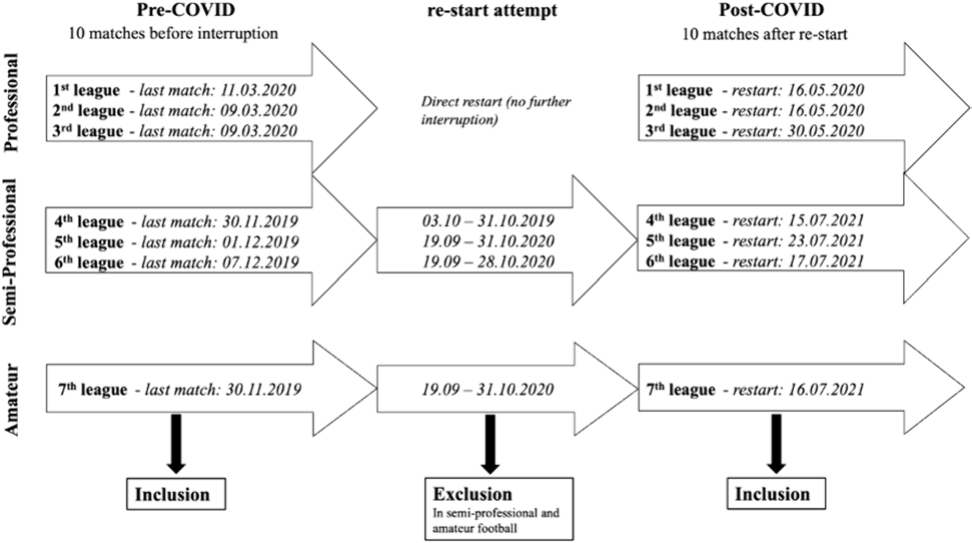
Overview on the study population with study period and levels of play

The COVID-19 pandemic, which began in 2019, led to a global lockdown and restrictions in all areas of life. During the lockdown period, social contacts were limited, and all national and international sporting events were banned. This ban also interrupted the course of the soccer season [15]. In amateur soccer, some leagues had to cancel more than half of their season matches. The German professional soccer leagues were among the first professional sports in the world to resume training and playing after a lockdown period of two months [15]. Strict hygiene regulations, rigorous testing, and limited contact were prerequisites for the quick restart. Amateur leagues resumed training and match activities at a later date, resulting in a longer period of training and playing restrictions (Fig. 1). Often, the only physical exercise for amateur soccer players is team training, and the impact of the COVID restrictions on overall physical and mental health has already been discussed elsewhere [18]. The effect of such prolonged training and match interruption on injury rates is of great scientific interest, particularly in the case of long-term injuries. Studies analyzing the contact times of players after the restart showed a significant reduction in close contact within a 2-meter radius as a possible effect of the lockdown [9, 22]. Up to 88% of injuries to the anterior cruciate ligament (ACL) are non-contact or indirect contact injuries (44% each), and ACL injury due to direct contact is much less common (12%) [5, 6, 21]. Irrespective of the injury mechanism, ACL injuries result in long periods of inactivity [23].

For professional players in Germany’s first league, the Bundesliga, the overall injury rate did not increase during lockdown compared with the rate before the pandemic [11]. However, there are no specific data on the incidence of ACL injuries. Furthermore, data on the impact of the interruption on amateur leagues are also lacking.

Therefore, the aim of this study was (1) to analyze the influence of the COVID-19 pandemic and the restriction of match and training activities on the occurrence of ACL injuries in the different soccer leagues and (2) simultaneously show possible changes in the injury mechanism due to reduced contact after the resumption of sports activities.

## Materials and methods

### Study population and design

This prospective registry study compares the incidence and patterns of ACL injuries in all professional men’s soccer clubs in Germany and all non-professional clubs of the Bavarian Football Association before and after the COVID-19 pandemic lockdown. Injury data have been recorded in a standardized manner in the prospective nationwide ACL registry in German soccer since the 2014–15 season [20]. The present analysis includes all injuries occurring between November 2019 and July 2021. All soccer players with a new ACL injury who had actively played in one of the German men’s professional leagues (1st-3rd men’s professional league) or in the Bavarian amateur leagues (4th–7th men’s amateur league) during the study period were included in the study population. All levels from the 1st to the 6th league and 8 of the 12 leagues of the 7th men’s soccer league were included for further analysis. Four leagues in the 7th league were excluded from analysis due to missing injury data. Semi-professional and amateur leagues attempted to resume match activities between September and October 2020. This period was excluded due to irregular training and match activities at the amateur levels (Fig. 1). Injuries were categorized as pre-COVID and post-COVID ACL injuries according to the date of injury (Fig. 1). The study population was divided into men’s professional (1st–3rd league), semi-professional (4th, and all 5th and 6th leagues), and amateur soccer (7th leagues). Professional soccer players were defined as full-time athletes with a contract, a salary, and full insurance; semi-professional soccer players were defined as paid players with a contract and insurance, but without sufficient income to live on.

### Injury documentation and data collection

Each year, soccer officials, clubs, and team physicians are invited to participate in the ACL registry in German soccer and to report each new ACL rupture to our study office. In parallel, the study team double-checks the registry data with national media data using a standardized data collection method to avoid typical data loss and to reduce the drop-out rate [20]. Krutsch et al. (2020) validated the double-check system for media analysis of ACL injuries in soccer, confirming 100% of injuries [10, 19]. Based on these ACL registry data, official match statistics were analyzed after each season for return-to-competition characteristics of the injured player. Thereby, the time of return-to-competition was determined by analyzing the official match records and by comparing it with the recorded date of injury. At the same time, match performance was assessed by recording the level of play, the total number of official matches played, and the number of minutes played. These data were recorded retrospectively for the season before and the season of the ACL rupture and prospectively for three seasons after the injury, providing a record of match performance over the course of five seasons.

Injury reporting for this study was adapted according to the commonly used injury reporting protocol in soccer established by Fuller et al. (2006) and according to previous epidemiologic injury studies by this study group [1, 8, 12, 13].

### Statistical analysis and data assessment

Categorial data are expressed as frequency counts (percentages) and continuous data as mean ± standard deviation (SD). Proportions between groups were compared with the Fisher’s exact test and continuous variables with the t test. Sample size was not calculated because the aim of the study was to recruit as many injured players as possible from the national and regional soccer associations for the analyzed seasons. The study office used the RedCap-System for data management and IBM SPSS Statistics, version 26.0, for data analysis. The study design of the National ACL Registry in Soccer was approved by the Ethics Committee of the University of Regensburg (ID: 22-2807-101).

## Results

In this analysis of the ACL registry in German soccer, 88 ACL ruptures were identified between November 30, 2019 and July 23, 2021. Four patients (4.5%) were excluded due to weak data and insufficient records between September 19 and October 31 2020. Overall, 84 amateur and professional soccer players were included for further analysis. Their anthropometric data are summarized in Table 1. In the pre-COVID population, 69.7% of injuries occurred in match situations, but this rate increased to 88.9% after the restart.

**Table 1:** Anthropometric data of the study population

	Pre-COVID (n = 40)	Post-COVID (n = 44)
	n ± SD (min; max)	n ± SD (min; max)
Age in years	24.7 ± 4.1 (19; 33)	23.4 ± 6.6 (18; 38)
Weight in kg	75.5 ± 7.7 (56; 96)	78.4 ± 5.8 (69; 92)
Height in cm	181.5 ± 6.6 (162; 194)	181.1 ± 4.7 (172; 193)
BMI in kg/m^2^	22.9 ± 1.9 (17.3; 25.5)	23.8 ± 1.2 (21.5; 26.3)
Experience in years	18.3 ± 4.8 (10; 27)	19.7 ± 6.0 (10; 33)
Level of play	n (%)	n (%)
Professional	5 (12.5)	4 (9.1)
Semi-professional	23 (57.5)	25 (56.8)
Amateur	12 (30.0)	15 (34.1)

**Fig. 2:**
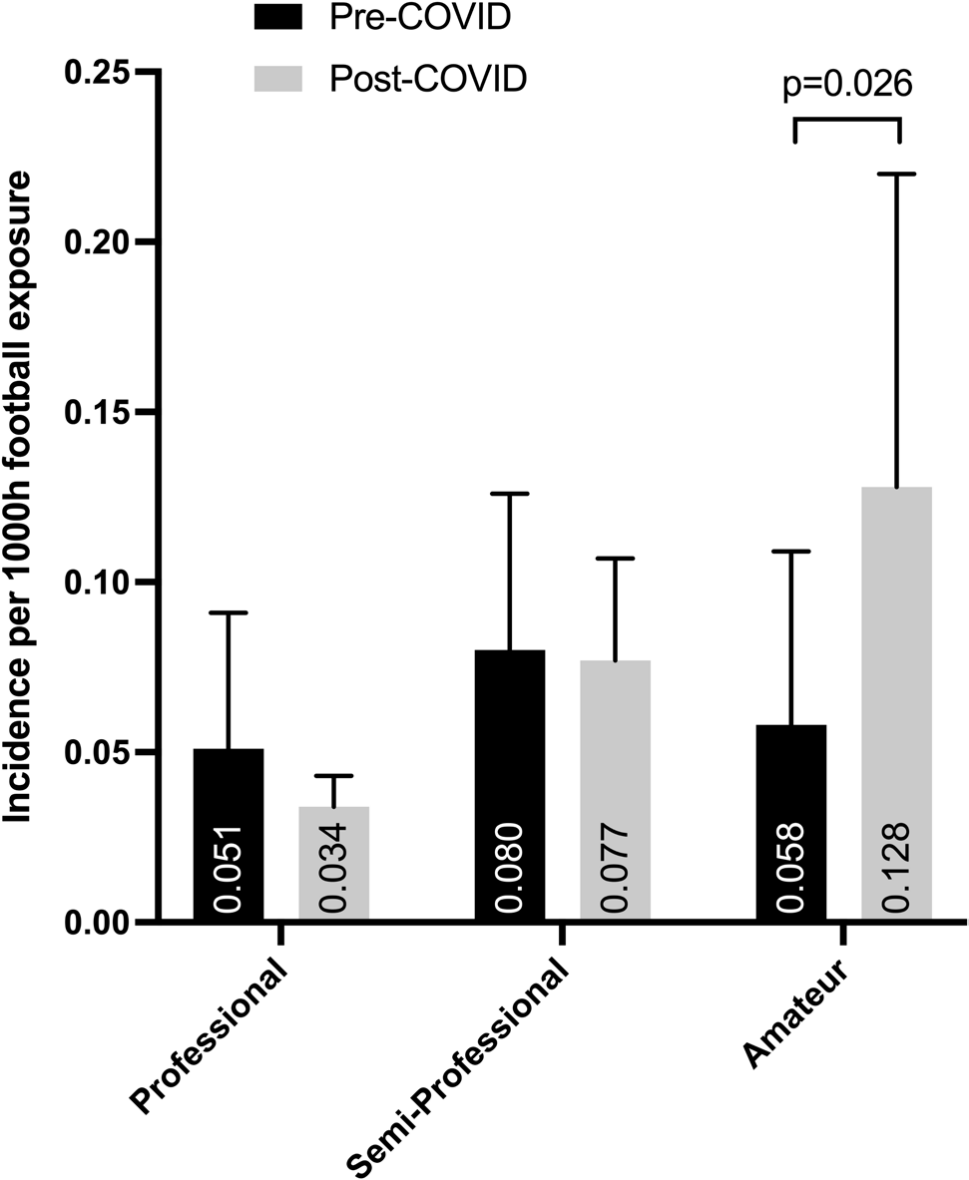
Incidence of ACL injuries in professional, semi-professional, and amateur soccer before (pre-COVID) and after (post-COVID) the COVID-19 pandemic lockdown

The overall incidence of ACL injuries was 0.081 per 1000 h of match and training exposure. The pre-COVID period showed an incidence of 0.083 per 1000 h (SD: 0.046) and the post-COVID period an incidence of 0.079 per 1000 h (SD: 0.044) (*p* = 0.699). In amateur soccer, the rate of ACL injuries significantly increased (*p* = 0.026), while in professional and semi-professional soccer, the incidence of ACL injuries decreased without statistically significance either professional (*p* = 0.436) or semi-professional (*p* = 0.802) soccer (Table 2; Fig. 2).

**Table 2:** Incidence of ACL injuries per 1000 h of match and training exposure in German soccer before and after the COVID-19 pandemic lockdown

	Pre-COVID	Post-COVID
Professional soccer	0.051 ± 0.040	0.034 ± 0.009
1st league	0.110	0.027
2nd league	0.027	0.027
3rd league	0.023	0.047
Semi-professional soccer	0.080 ± 0.046	0.077 ± 0.030
4th league	0.0	0.110
5th league	0.106	0.121
6th league	0.088	0.053
Amateur soccer	0.058 ± 0.051	0.128 ± 0.092

**Fig. 3:**
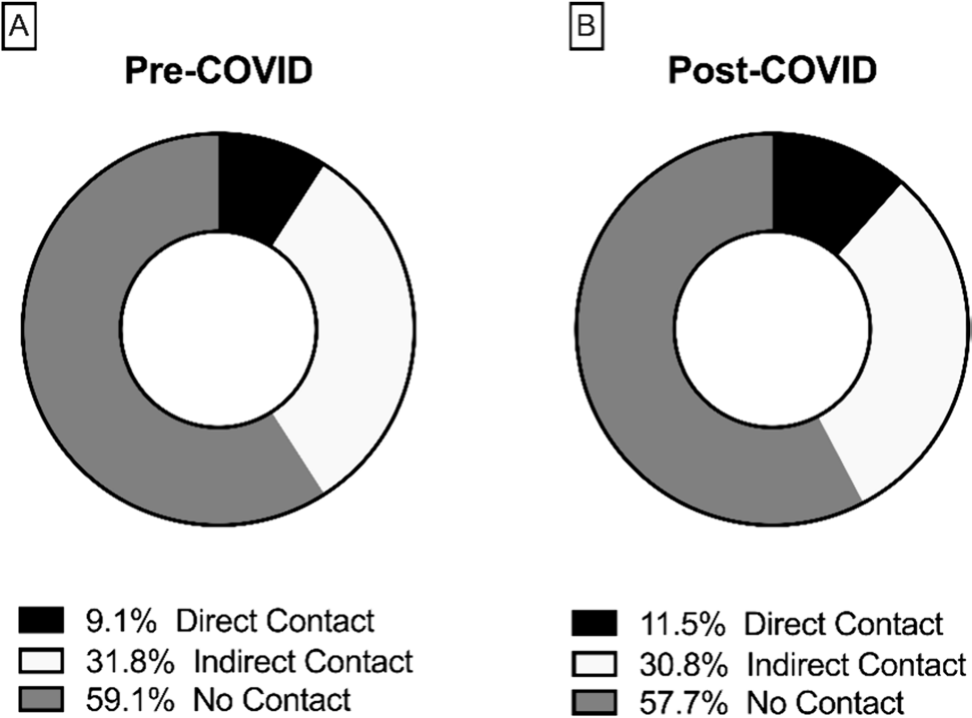
Percentage of ACL injury mechanism before (**A**) and after (**B**) the COVID-19 pandemic lockdown

ACL injuries in the pre-COVID population were predominantly non-contact (59.1%) and indirect contact injuries (31.8%). After the restart of all soccer leagues, 57.7% of ACL injuries were identified as non-contact and 30.8% as indirect contact injury. No fouls by the opponent were reported in the pre-COVID period and three fouls in the post-COVID period (Fig. 3).

## Discussion

The main finding of this investigation was a significantly increased incidence of ACL injury in amateur soccer (*p* = 0.026), whereas no change was reported in the semi-professional and professional soccer leagues. Secondly, the ACL injury mechanism in the post-COVID period did not significantly differ from that in the pre-COVID seasons and was dominated by the non-contact injury mechanism (pre-COVID: 59.1%; post-COVID: 57.7%).

Due to the unprecedented disruption of daily life, including the ban on all team sports, the period after the resumption of play is still under close scientific scrutiny. Reporting from the UEFA Elite Club Injury Study, Waldén et al. (2022) described no increased incidence in match injury and injury burden in professional men’s soccer after the lockdown period but an increased incidence in training injuries [24]. When investigating the first men’s soccer league in Italy, Marotta et al. (2021) found no significant increase in muscle injuries in the post-COVID period [14]. Krutsch et al. (2022) also found no significant increase in total injuries in German professional men’s soccer after the lockdown period [11]. Changes in injury incidences were not only investigated in professional soccer but also in the semi-professional and amateur leagues, albeit much less frequently. Ross et al. (2022) reported no significant difference in the incidence of ACL injuries, but a rapid 26% increase in total injuries and a 45% increase in joint injuries in the first month after the lockdown period. The authors concluded that semi-professional and amateur players had a higher risk of joint injury, especially in the first few months of training after the lockdown period [17]. Keeping this in mind, there are definitely parallels to the results of the current study with regard to pre- and post-COVID ACL injuries. According to the ACL registry in German soccer, there was no significant increase in ACL injuries among professional and semi-professional soccer players (professional: *p* = 0.436; semi-professional: *p* = 0.802). In amateur leagues, however, the incidence of ACL injuries increased significantly (*p* = 0.026). This difference can be explained by several factors. First, the lengths of the mandatory break from training and match activities differed between the leagues depending on the level of performance. While the first and second German soccer leagues returned to play after only about two months, the downtime in amateur sport was much longer (approximately 19 months, see Fig. 1). Second, professional and amateur soccer players had very different training options during the break, when no team training sessions were allowed. Schüttler et al. (2022) investigated the training sessions of professional and amateur soccer players during lockdown [18]. For both groups of players, the COVID-19 lockdown understandably affected their soccer training, especially training concepts with the ball, which resulted in changes in the exercise load. The authors concluded that muscular load may affect the susceptibility to injuries, especially of amateur players due to the lack of training supervision and professional training schedules. Nevertheless, even the replacement training for professional soccer players at home led to a decrease in explosive power and speed performance due to the change in training location and techniques. Friebe et al. (2022) showed that general strength and endurance could still be maintained through workouts at home [7]. In conclusion, not only was the training break longer for amateurs, but also the resumed replacement training, if any, was not comparable to the structured, adapted training concept for return-to-play in professional soccer. These factors may explain the increase in ACL ruptures in amateur soccer after the end of the lockdown. Conversely, the lack of increase in the incidence of ACL injury after return-to-play in professional soccer can also be seen as a success of the preventive approaches of replacement training and early return-to-competition after the ban. Apart from that, in retrospect, the re-start of German professional soccer can also be considered safe from an infectious disease point of view [15]. Finally, when considering the incidence of ACL injuries in other professional sports, several studies, for instance in American football, showed that the incidence of ACL injuries remained constant after the lockdown period. Only the timing shifted from pre-season to season [2, 16].

This study not only investigated the incidence of ACL injuries but also possible changes in the mechanism of ACL injuries. Dominant biomechanical factors, especially for the non-contact ACL injury mechanism, are the valgus knee during pivoting and cutting maneuvers as well as the landing phase after jumping combined with an axial compression force [3, 4].

As mentioned above, ACL injuries often occur as non-contact (44%) or indirect contact (44%) injuries [6]. This study showed a similar distribution in the mechanism of ACL injuries for both the pre- and post-COVID periods (see Fig. 3). According to the ACL registry in German soccer, there were no significant changes in the ACL injury mechanism. Szymski et al. (2023) recently showed that the pandemic resulted in a 25% reduction in match-specific contacts [22]. No change in contact patterns was observed, possibly because ACL injuries are rarely caused by direct contact.

### Limitations

Despite its strengths, the present study has limitations that should be taken into consideration when interpreting its results. First, one limitation is the exclusion of data collected at the amateur and semi-professional levels between September and October 2020. An attempt was made to restart match activities during this period, but due to irregular training and match activities at the amateur level, this period could not be included in the survey, which may be considered a confounder. Second, ACL injury is a relatively rare but severe injury; therefore, the study population was limited and thus also the power of the conclusions. Third, the content of the training sessions in the semi-professional and amateur leagues is not well documented. It would be helpful to know the training regimens and the extent to which players followed them during lockdown to better understand the increase in ACL injuries among amateur players after the lockdown period and to compare the results with those of semi-professional and professional players. Finally, media-based studies are associated with general limitations that may lead to inaccurate injury statistics and questionable conclusions. These limitations include, for example, incomplete data records leading to exclusions. Nevertheless, the example of ACL injuries has shown that media analyses provide sufficient and reliable data [11, 19]. The ACL registry in German soccer in particular is characterized by a well-defined methodology and an accurate differentiation between weak and valid information. These methods provide media-based data with high validity, which distinguishes the present study from other media-based injury incidence reports.

## Conclusion

The study demonstrated in an analysis of the German ACL registry an increase of ACL injury incidence in amateur football after the lockdown. In professional and semi-professional athletes a decrease of the incidence was reported, however without statistical significant results. Possible reason for this trend in different levels of play were the longer lockdown in amateur football and lack of training during the lockdown. The ACL injury mechanism did not change from the pre- to the post-COVID period either in professional or amateur football. Further research is needed to identify the factors that increased the incidence of ACL injuries in amateur soccer players after the COVID-19 pandemic lockdown and how this incidence may evolve in coming post-COVID seasons.

## Supplementary Information

Below is the link to the electronic supplementary material.

Supplementary material 1 (DOCX 34.1 kb)
